# Women’s Experiences and Preferences in Relation to Infertility Counselling: A Multifaith Dialogue 

**Published:** 2011-12-22

**Authors:** Robab Latifnejad Roudsari, Helen T. Allan

**Affiliations:** 1 Department of Midwifery, School of Nursing and Midwifery, Mashhad University of Medical Sciences, Mashhad, Iran; 2 Division of Health and Social Care, Faculty of Health and Medical Sciences, University of Surrey, Guildford, UK

**Keywords:** Infertility, Counselling, Religion, Spirituality, Experience

## Abstract

**Background:**

Religion and spirituality are a fundamental part of culture and influence how individuals experience and interpret infertility counselling. Thus far, little research has examined the influence of religiosity on the experience of infertility, and to our knowledge no study exists investigating the responses of religious infertile women to counselling. In this study we explored Muslim and Christian women’s experiences and preferences with regard to infertility counselling.

**Materials and Methods:**

Using a grounded theory approach, 30 infertile women affiliated to different denominations of Islam (Shiite and Sunni) and Christianity (Protestantism, Catholicism, Orthodoxies) were interviewed. Data were collected through semi-structured in-depth interviews at fertility clinics in the UK and Iran, and analyzed using the Straussian mode of grounded theory.

**Results:**

Emerging categories included: Appraising the meaning of infertility religiously, applying religious coping strategies, and gaining a faith-based strength. These were encompassed in the core category of ‘relying on a higher being’. Religious infertile women experienced infertility as an enriching experience for spiritual growth. This perspective helped them to acquire a feeling of self- confidence and strength to manage their emotions. Hence, they relied more on their own religious coping strategies and less on formal support resources like counselling services. However, they expected counsellors to be open to taking time to discuss their spiritual concerns in counselling sessions.

**Conclusion:**

In addition to focusing on clients’ psychosocial needs, infertility counsellors should also consider religious and spiritual issues. Establishing a sympathetic and accepting relationship with infertile women will allow them to discuss their religious perspectives, which consequently may enhance their usage of counselling services.

## Introduction

Infertility is recognized around the world as a distressing experience with the potential for threatening individual, marital, family and social stability (1). Individuals suffering from infertility will confront with complex issues including biological, psychological, therapeutic and ethical dilemmas. Discussion of these concerns in a counselling context is often beneficial for patients (2). Counselling is “a process through which infertile couples are given the opportunity to explore themselves, their thoughts, feelings and beliefs in order to come to a greater understanding of their present situation and also to discover and clarify ways of living more satisfyingly and resourcefully” (3). Counselling with infertile individuals is often about support, advice, guidance and the clarification of life goals.

The aims of infertility counselling therefore are to explore, understand and resolve issues arising from infertility and infertility treatment, and to clarify ways of dealing with the problem more effectively (2). Thorn argues that counselling can contribute to improving psychological and social health as well as helping to minimize drop-out rates in treatment (4). The human fertilization and embryology authority (HFEA) which regulates assisted reproduction in the UK has stipulated that psychosocial counselling must be offered to any patient seeking *in vitro* fertilization (IVF) or donor insemination. As described in the HFEA code of practice, the purpose of counselling is to provide patients with emotional support in times of crisis, and to help them come to terms with their treatment choice and its effect on their lives (5).

Also the UK Department of Health evidencebased clinical practice guideline has mentioned that the government is aware of the evidence of the benefits of counselling and believes that it can play a valuable role in helping patients make informed reproductive decisions and understand their implications (6). The importance of counselling has been acknowledged in laws concerning human reproductive technologies (7). The appointment of at least one member of staff to fulfil the role of counsellor has appeared in the Human Fertilization & Embryology Authority code of practice as well. All licensed IVF clinics in the UK are required to offer patients counselling (8). The National Health and Medical Research Council (NHMRC) of the Australian Federal Government has also stipulated the right of donor-conceived people to be informed of their biological origins; and the provision of comprehensive counselling about the social, psychological, physical, ethical, financial and legal implications of third-party reproduction to those considering donating or receiving gametes or embryos and entering surrogacy arrangements (9).

Despite the aforementioned agreements on the necessity of counselling infertile couples, very few patients use these services when made available to them (10). Prospective (11) and retrospective studies (12) have also shown that only about 18-21% of patients who have been offered counselling decided to attend individual or couple counselling sessions. Marcus et al., in an internet- based survey on 244 users of an independent infertility website found that 73% of all couples were offered, or obliged, to receive counselling compared to 91% of those patients treated in the UK. Of the patients who took part in the survey, only 30% received counselling (8).

The reasons for not using psychosocial counselling services have been investigated by Boivin et al. (1999) (10). They found that three main factors contribute to low uptake of psychosocial counselling, including 1. patients’ comfort level with the counselling, 2. coping resources available to the patients and their ability to manage the strains of infertility and, 3. practical concerns for arranging a meeting with counsellors. Emery et al. indicated that infertile women who refused counselling mostly cited the difficulty of taking time off from work or their lack of interest because they “felt strong enough” (13). Marcus et al., also reported that in patients who did not receive counselling, the main reasons were: “felt I can cope on my own” (37%), “counselling was not offered” (21%), and “did not think it would be beneficial” (15%) (8).

The reliance on one’s ability and resources, which has been reported by infertile patients in the aforementioned literature, could be discussed in relation to individuals’ religion and spirituality. Religion is a particular doctrinal framework which guides sacred beliefs and practices about a higher power or God. It is a system of beliefs and practices that structure how people worship. Spirituality refers to the beliefs and practices that connect people with sacred and meaningful entities beyond themselves. These beliefs and practices often create a relationship with a supreme power which gives meaning and purpose to life (14, 15). Religion may be both a resource and a burden. For some infertile couples, religion may provide opportunities to maintain hope and give meaning to their experiences of suffering and loss inherent in the infertility experience. However, faced with existential dilemmas, psychological distress and social stigma, they may experience a ‘crisis of faith’ or alternatively, find peace and comfort in their faith community and/or its rituals that help them meet the challenges of infertility (16).

Latifnejad Roudsari et al. have argued that infertile women turning their attention to religious and spiritual beliefs show connectedness to a higher being who can be trusted and believed, as a source of strength, guidance, and support (17). They endeavour to maintain, develop, and renew this relationship to be able to deal with the hardship of infertility. Latifnejad Roudsari et al. have discussed that women’s views on socialization as a religious value motivate them to search reassurance through the love and care of congregation (17). Having this unique worldview, infertile women give sacred meaning to life and talk about an internal knowing, certainty and assurance that they will be blessed by God (18). This confidence may impact their decisions regarding seeking therapeutic approaches, including the usage of counselling services.

In this regard, Hynie and Burns have argued that religious beliefs may provide limits on the acceptability of various treatment options (16). Hence, infertility counsellors, in providing a suitable approach, should consider religion as a potential asset as well as a potential liability for infertile couples. They must be aware of the impact of faith and religion on the infertile couple and how religion is, for them, either a benefit or a burden. Thorn argues that much of the current debate in the area of counselling is based on Western values and assumptions; however, couples’ management strategies regarding the psychosocial implications of infertility can only be understood in the context of their specific culture and religion. He emphasized that sharing professional experience on an international level can contribute to further professionalization and increased awareness of cultural differences in this area (19).

Thus far, little research has examined the influence of religiosity on the experience of infertility (17, 18, 20-22) and to our knowledge, no study exists investigating the response of religious and spiritual infertile women to counselling and also how useful they may find it. The purpose of this qualitative study was to explore Muslim and Christian infertile women’s experiences and preferences concerning counselling and their main rationales for opting to receive or not to receive counselling.

## Materials and Methods

Grounded theory was the methodological approach that underpinned and guided this research. Grounded theory is a qualitative research method that is useful for generating research-based knowledge about the behavioural patterns that shape social processes as people interact with each other (23). It makes its greatest contribution in areas where little research has been done. Grounded theory systematically applies specific procedural steps to ultimately develop a theoretically complete explanation about a particular phenomenon (24).

The study settings were two referral hospitals in London and one Iranian Infertility Research Centre in Mashhad. To explore the experiences of infertile women regarding counselling in a wider religious context and a larger ethnic mixture it was decided to recruit the participants from the multifaith society of the UK and the religious community of Iran.

Participants were thirty infertile women with primary or secondary infertility, who had been diagnosed as infertile through preliminary tests by their general practitioners. Women who were infertile due to a physiological problem in the female, male or both and those who were infertile due to unknown causes, irrespective of the duration for which they had been trying to become pregnant, were recruited. Women with an adopted child or with a newly positive pregnancy test, who no longer struggled with fertility problems during the study period, were excluded from the study. In addition, women who might not adequately understand verbal explanations or written information given in English were not included in the study.

Participants were affiliated to different denominations of two monotheistic religions, i.e. Islam (six Shiites and six Sunnis), and Christianity (ten Protestants, six Catholics and two Orthodoxies). Having chosen grounded theory as the methodological approach, the sample size was determined by purposive and theoretical sampling and data saturation. To maintain diversity in participants’ recruitment it was endeavoured to consider the diversity in terms of participants’ age, social class and ethnic background, in addition to their religious affiliations. A summary profile of all participants has been demonstrated in table 1. To recruit women, they were given an invitation letter and the patient information sheet. They were given time to study the information sheet and the opportunity to ask questions from the researcher. If they were interested in taking part in the study, they were interviewed after their appointment, or alternatively an appointment was arranged with the researcher in the future.

In this study a combination of data sources including formal interview, observation of nonverbal behaviours during the interviews and the writing of post-interview notes and diaries, were used to collect the data. All interviews were conducted by the first author who is a midwife lecturer/ researcher with experience in interviewing infertile women. She was trained at the University of Surrey, UK as a qualitative researcher and supervised by an experienced qualitative researcher (the second author).

Interviews were conducted face-to-face in one of the interview rooms of the fertility clinics allocated for this purpose, using a semi-structured interview guide. In order to keep the natural flow of the dialogue between the participants and researcher, the questions about religion and spirituality were introduced when the interviewee spontaneously mentioned God, religion or spirituality as a resource for managing emotions. If no reference to God, religiosity or spirituality was made by the participant, then the researcher asked the related questions at the end of the interview. Participants were asked about how their religious and spiritual beliefs may affect the way they perceive infertility, the strategies that they use for coping with their fertility problem and their viewpoints on getting help from counselling services. Each interview took on average between 45 and 60 minutes and was audio-taped and transcribed fully for data analysis. It is noteworthy that the interviews related to the Iranian participants were carried out in Persian. Then after data transcription they were translated from Persian to English by the first author whose native language is in the same dialect of the interviewees, and who had been trained for this purpose at Language Centre, University of Surrey, UK. This could minimize inherent threats to the validity of cross-language translation. To validate the translation, two translated interviews were also checked by two native Persian speakers who were experts in English in Iran, as well as two native English academics in the UK.

Data analysis was accomplished adopting Strauss and Corbin’s mode of grounded theory that included three levels of open, axial and selective coding. It was concurrently carried out by data collection, i.e. the data collected were transcribed and analyzed immediately after each interview. One reason for this practice was that in grounded theory the incoming information from participants determines the information which should be sought. For quality assurance of transcription, transcripts were reviewed by both two researchers. Using the constant comparative method, data were coded line by line. Codes were grouped together in categories and the constant comparison of categories continued as the properties of each category emerged. As comparison went on, hypotheses about the relationships among concepts were generated and checked against raw data. To avoid potential misrepresentation of data and in order to seek consensus between the researchers, the codes, subcategories and categories were further discussed, which led to refinement of the scheme of abstracted categories.

Lincoln and Guba’s key concepts of rigour including credibility, conformability and transferability were used to support the enhancement of data analysis quality (25). In order to ensure that the phenomenon was investigated accurately, participants were permitted to guide the interview process and their own language and actual words were used at all levels of coding. Furthermore, prolonged engagement with participants for extended periods of time, member check in which respondents were asked to confirm findings, and peer debriefing were used to enhance the trustworthiness and credibility of the findings. To achieve transferability, strategies like thick descriptions and purposive sampling were used.

**Table 1:** Frequency distribution of participants’ demographic characteristics


Age (year)		Marital status		Education		Occupation		Ethnicity	

**< 30**	6	Married	27	High school	12	Employed	27	White British	5
**30-34**	10	Partnership	3	College	3	Housewife	3	Asian	7
**35-40**	11			University	15			African	6
**≥ 40**	3							European	4
								Asian British	4
								African British	4

Religion		Religious/Spiritual score		Infertility factor		Infertility duration		Type of infertility	

**Shiite/ Muslim**	6	14-33		Female	19	<5 years	16	Primary	26
**Sunni/ Muslim**	6	34-52	1	Male	4	5 to 10 years	3	Secondary	4
**Protestant/Christian**	10	53-70	4	Both	3	≥10 years	11		
**Catholic/ Christian**	6		25	Unknown	4				
**Orthodox/ Christian**	2								


The study was approved by Research Ethics Committees of Queen Charlotte’s and Chelsea Hospital, Elizabeth Garrett Anderson and Obstetrical Hospital and University of Surrey. All participants signed the informed consent form and were assured that anonymity and confidentiality would be maintained. They could refuse to participate or withdraw from the study at any time without prejudice to their clinical treatment.

## Results

Through analysis three categories emerged including: appraising the meaning of infertility religiously, applying religious coping strategies, and gaining a faith-based strength. These were encompassed in the core category of ‘relying on a higher being’. The core concept was that the majority of religious infertile women believed in a supreme power, who can be called to for assistance in the occasion of devastation and desperation in their lives. This made the experiences of infertile women who were affiliated to different religious faiths, congruent and well-matched.

### Appraising the meaning of infertility religiously

The findings of this study showed that participants using a religious/spiritual meaningmaking framework tried to reappraise their illness religiously and spiritually. They trusted a higher power who can protect individuals, and endeavoured to gradually accept themselves as infertile. They gave a sacred meaning to everything in their life, had a particular loving relationship with God and considered every Godgiven phenomenon as a gift, believing in the logic behind it. They viewed their infertility as God’s will and believed that nothing can happen without God’s contribution as He has absolute control over people’s lives; thus people should accept what God has decreed for them. In addition to this, participants in the current study talked about an internal knowing, certainty and assurance that they will be blessed by a compassionate and merciful God, either through having a child or in other ways as *“God never let them down and if he doesn't give them one thing, He gives them something else”* (NA/ 28 Y./ Shiite Muslim/ Asian).

They contemplated that they should accept God’s plan with enthusiasm as His will is the most advantageous course for their lives, because they are being loved by God and He knows *“what’s best really”*. Moreover, the findings suggested that religious participants, due to having a *“bigger understanding of life”* did not view infertility as *“just trying to have a baby”*. They were hopeful that infertility would be a *“life-enriching experience”* and a *“positive process”* to enhance their *“reliance on God”* and to improve their *“spiritual growth”*.

### Applying religious coping strategies

This worldview resulted in optimism and positive thinking which empowered the women in their journey to be able to accept their identity as infertile. Gradually they tried to take responsibility and control over all aspects of their lives by adopting some strategies to cope with infertility. They employed a wide spectrum of religious coping strategies, which are rooted in their religious teachings. These strategies consisted of a combination of positive and negative religious coping strategies which enhanced their emotional capability and as a consequence helped them to overcome their stressful situation.

Positive religious coping strategies included benevolent religious reappraisal: *“Religion absolutely gives me the strength to deal with infertility”* (SR/ 33 Y. / Sunni Muslim/ Asian British), belief in spiritual support: *“If you ask anything from Him (God) He will give you”* (NA/ 28 Y./ Shiite Muslim/ Asian), engagement in rituals: *“I do my prayer, so I cope with things”*, (AH/ 32 Y./ Christian: Church of England/ White British), belief in miracles: *“I think people’s belief in miracle gives them spiritual help”* (RJ/ 38 Y./ Christian: Orthodox/ European), belief in timing: *“Whenever the time is right we will have a child”* (HA/ 26 Y. / Sunni Muslim/ Asian British), and seeking support from congregation and clergy: *“A lot of my good friends know and they pray for us”* (AH/ 32 Y./ Christian: Church of England/ White British).

In the other hand, some religious participants adopted negative religious coping strategies including demonic reappraisal:* “There is something inside the people living with them known as Jinn who blocks everything *(IM/ 30 Y./Sunni Muslim/ African), spiritual discontent: *“Occasionally I complain to God and say O’ God if really you are present everywhere why you don't respond me when I cry and ask you?!”* (NA/ 28 Y./ Shiite Muslim/ Asian) and discontent with clergy: *“Priests’ answers regarding using donor procedures wouldn't affect my decision” *(CA/ 38 Y./ Christian: Catholic/ White British).

A range of non-religious coping strategies such as ignorance: *“There is always the element that it might not happen but I don't want to think about that”* (AH/ 32 Y./ Christian: Church of England/ White British), minimization:* “I think at the end of the day, it’s not dying; it’s just having a child”* (HA/ 26 Y. / Sunni Muslim/ Asian British), and compensation: *“I would like to get more success in other aspects of my life which can cover my inability to get children”* (NA/ 28 Y./ Shiite Muslim/ Asian).

Having adopted these varieties of coping resources helped infertile women to obtain a feeling of self-confidence and empowerment and consequently the ability to manage their emotions. One of the Baptist participants commented: *“I think I’m a strong person and I can cope with the situation, so I don’t need somebody else to help me”* (ED/ 40 Y./ Christian: Baptist/ European).

### Gaining a faith-based strength

The other issue that the majority of the religious participants discussed was the sufficiency of their religious teachings as the best source of counselling: *“I think my religion is the best counselling I can be given” *(HA/ 26 Y. / Sunni Muslim/ Asian British). They thought that they did not need any emotional and/ or psychological support provided by counsellors. This concept was clearly expressed by one of the Muslim women: *“Emotionally I am OK; thanks to God it has not affected me and I think I am psychologist of myself, I don’t need to get help apart from my Allah, my God”* (SR/ 33 Y. / Sunni Muslim/ Asian British).

They believed that they were able to find everything in their holy book: *“I don’t go to counselling and I just think if you are religiously committed you don’t need any of these things; because we’ve got all it in our holy book”* (IM/ 30 Y. / Sunni Muslim/ African). Some participants emphasized their religious consciousness and their knowledge about the purpose of life. They pointed out that their faith has taught them how to manage life, so they did not feel the need for any sort of counselling. One of the Muslim participants indicated: *“I know what the faith is, the meaning of things, if something can help you, the way you could run the life, everything I know and I don't need to go there (counselling) and I never go there”* (IM/ 30 Y. / Sunni Muslim/ African). A Christian participant (Church of England) stated that her faith has given her the strength and capability to handle situations throughout her life:* “I am able to hold it in perspective to the rest of my life and that’s what faith does”* (AH/ 32 Y./ Christian: Church of England/ White British).

It is worthwhile to say that some of the religious participants were even eager to help other people struggling with fertility problems, and it showed their emotional strength. In this regard, one Baptist participant said* “I think I can help the other people who are in the same situation like me and I don’t need somebody else to help me. I’m just thinking of couples who can’t cope with the situation, to support them, to encourage and to help them to find a solution for their problem”* (ED/ 40 Y./ Christian: Baptist/ European).

In contrast, some religious infertile women acknowledged counsellors’ help and support, but they liked religious issues to be addressed in their counselling sessions. One of the Christian participants (Church of England) indicated her hesitancy in choosing either somebody who is an expert in infertility counselling or someone who is a strong religious person but with less expertise. Nevertheless, her preference in both cases was having the opportunity to talk about God, because she believed that life does not make sense for her without God: *“The counsellors often help, but in my experience I want to talk about God, bring God in, because life doesn’t mean to me, life doesn’t make sense without God”* (AH/ 32 Y./ Christian: Church of England/ White British).

## Discussion

Research studies have shown that religion and spirituality are highly valuable for many people during their confrontation with crisis, trauma and grief (20- 22). There is an increasing awareness among medical and mental health caregivers that spiritual well-being is an important dimension to physical and emotional health and that there is a generally positive relationship between religious involvement and health outcomes (26). For this reason, over the past several years there has been an expanded body of literature on integrating religion and spirituality into clinical practice.

However, little research has examined the influence of religious beliefs on the experience of infertility and patients’ decisions regarding its treatment. Also, very little is known about considering religious concepts in infertility counselling. Molock who has investigated the religious and cultural aspects of infertility in the African- American community argues that spirituality is a very important cultural value for African Americans (27). For this reason, Molock suggests that during the initial stages of counselling it is important to note how salient religious practices are in the client’s life (28). She emphasizes that it is also important to ask clients about their understanding of infertility not from a medical standpoint but on a “personal level”. Dutney has argued that infertile patients who are religiously active are probably in a process of reframing their faith in the light of their experience of infertility (20).

The findings of this study showed how religious and spiritual frames of reference transformed infertile women’s views of infertility from an unbearable life crisis to a tolerable process which can be dealt with in order to achieve spiritual growth and development. This notion is congruent with what Sewpaul mentioned regarding infertile women who reappraised infertility as an opportunity for re-evaluation of one’s life, values and relationship with God and as a challenge which provided the opportunity for positive change (22). We argue that these kinds of positive reappraisals of infertility by the majority of religious infertile women give them self-empowerment and self-worth in their journey to be able to confront infertility with less difficulty and to accept their identity as infertile (29).

The findings of this study highlighted that religious participants achieved feelings of optimism and peace regarding the emotional burden of infertility by adopting religious/ spiritual coping strategies, which arise from their religious teachings and divine outlook on life. This finding is in agreement with what Domar et al. observed (21). In a quantitative study exploring the role of religiosity and/or spirituality in shaping the subjective psychological well-being of infertile women, they found that infertile women with higher levels of spiritual well-being reported fewer depressive symptoms and less overall distress from their infertility experience. They suggested a relationship between spirituality and the psychological well-being of women undergoing infertility treatment.

A further finding of this study was that most religious infertile women felt that their religious coping resources were sufficient to manage the strain of their infertility. They experienced their religious teachings and holy book as the best source of counselling and believed that they have religiously been taught how to manage life crises. Also the support that they received through their religious husbands, congregation and clergies resulted in less reliance on formal support resources like counselling services. Boivin et al. in her study entitled: *“Why are infertile patients not using psychosocial counselling?”* has similarly discussed that the majority of infertile women have been able to receive good help from informal sources of support, i.e. spouse, family and friends (10). They therefore did not consider themselves distressed to the point of needing counselling services and felt that counselling would not actually help them to cope with infertility. De Klerk et al. have also reported that there is little perceived need for psychosocial counselling by infertile couples who are in stable relationships and benefit from other sources of support available to them, like family or friends (30). Boivin has argued that despite the best efforts of counsellors, some highly distressed patients will refuse to attend counselling, because they do not recognize the need for such help (31). Molock in this regard, has discussed that counsellors need to be sensitive to this issue that many religious people are uncomfortable venturing into counselling because of 1. their tendency to distrust disclosing personal information to “strangers” and preference to resolve their “emotional” problems through their family, friends and clergies in their community 2. the stigma attached to seeking mental health services and 3. feeling uncomfortable discussing issues concerning sexual behavior due to the close association between infertility and sexuality (28).

It is worthwhile to point out that in this study some of the participants expressed their desire and wish for religious and spiritual topics to be addressed in counselling. Puchalski, with regard to paying attention to the spiritual concerns of patients, has argued that spirituality may be a dynamic force in the patients’ understanding of illness and can affect their decision-making for treatment. Therefore an understanding of patients’ spirituality is integral to their whole care (32). Eck discusses research which emphasizes clients’ preference for including their belief system in therapy (33). He cites Quackenbos et al. who reported that 78% of clients feel that religious values should be discussed in counselling (2000: 268). Worthington et al. also argued that clients who identify themselves as religious prefer religious themes to be discussed in their counselling (34). The guideline for good practice in infertility counselling provided by the British Infertility Counselling Association (BICA) takes account of the client’s cultural and faith context (2.2, P.1) and also the ethical, cultural, social and faith issues raised by assisted conception treatments and research (7.4, P.8) (3). In the guideline for counselling in infertility which has been written in collaboration with the UK, Germany, Spain, Belgium, Switzerland and New Zealand, in the section which exclusively deals with third party reproduction (gamete and embryo donation and surrogacy) it has been recommended to discuss religious and cultural considerations (35).

Latifnejad Roudsari et al. have argued that health professionals can encourage patients to initiate discussion regarding their religious and spiritual background, in addition to their medical history (36). In relation to addressing religious issues, Molock has commented that it is helpful to explore with clients how their spirituality helps and impedes their ability to cope with infertility (28). She has argued that if therapists are uncomfortable discussing spiritual issues, they should offer a referral to a pastoral counsellor who has been trained to address both spiritual and psychotherapeutic issues. They can also encourage support groups in particular religious communities. Dutney has also advised medical personnel to consider identifying people in the community to whom infertile patients can be referred for spiritual counselling, including ministers or priests who have personal knowledge of the experience of infertility (20). Molock indicates that it is important that counsellors address how they feel about their own spirituality, although this might be difficult for most of the therapists, as they have been trained to avoid religious issues in counselling. She emphasized, however, that it is important for infertility counsellors to appreciate that infertility is experienced as a spiritual crisis by many clients (28).

In the process of exploring the religious and spiritual experiences of infertile women, it is important to keep the limitations of this study in mind. One potential limitation was the researcher’s reliance on participants’ self-reports of their religiosity and spirituality. The other issue was the relatively small sample size which is a common issue in qualitative methodologies. However, although the sample size was small, it was purposeful and the logic and power of purposeful sampling lies in selecting information-rich cases for in-depth study. Furthermore, in qualitative studies the researchers do not attempt to generalize their explanations in an empirical way; instead, they try to make a theoretical generalization which is more productive.

## Conclusion

As infertility is a multifaceted problem and results in multiple losses, health professionals who are working in fertility clinics need to consider all aspects of holistic care when caring for women with fertility problems. Holistic care considers not only the psychological, social and cultural needs of individuals, but also their religious and spiritual needs (36). The findings of this study can enrich both medical and psychosocial professionals’ awareness and understanding of religious/ spiritual infertile women’s conceptualization of their illness (37). We argue that infertility counselling, in addition to focusing on the psychosocial needs of infertile couples, should also consider their religious and spiritual concerns, although this issue requires further research in relation to the different religious faiths. Molock, in this regard, argues that infertility counsellors must have knowledge of religious/ spiritual values and should consider these issues in the provision of psychotherapeutic interventions for couples experiencing infertility (28). We argue that providing infertility counselling in a religiously sensitive context can encourage religious/ spiritual infertile women to use counselling services and can help them to cope better with their stressful situation. Additionally, it can give a more holistic approach to infertility counselling and help infertile women better come to terms with their experiences.
